# Fractographic analysis of 3D printed hybrid ceramic single crowns with different aging and post-curing times: an in-vitro study

**DOI:** 10.1038/s41598-025-26531-x

**Published:** 2025-12-26

**Authors:** Salma Hatem, Bassem Emad, Nermeen Nagi, Aliaa Ibrahim Mahrous

**Affiliations:** 1 Royal College of surgeons of Edinburgh, University of Edinburgh, Edinburgh, Egypt; 2 Fixed Prosthodontics Faculty of Dentistry, Galala University, Galala city, Egypt; 3 Fixed Prosthodontics Department, Faculty of Dentistry, Fayoum University, Fayoum, Egypt; 4 Prosthodontic Department Faculty of Dentistry, MSA University, Cairo, Egypt

**Keywords:** Health care, Materials science, Medical research

## Abstract

Since increasing 3D-printed ceramics are being introduced to restorative dentistry, a critical evaluation of fabrication parameters for optimal clinical performance is necessary. While post-curing treatments have some influence on material properties, their impact on fracture resistance is not clear. This in vitro study aimed to systematically evaluate the effects of different post-curing times (10 vs. 20 min) and thermocycling aging (5,000 vs. 10,000 cycles) on the fracture resistance and failure modes of 3D-printed hybrid ceramic crowns (Saremco CrownTec). CAD software was utilized to create a standardized digital model of a mandibular first premolar crown. Eighty identical samples were produced with a high-precision 3D printer (Asiga Max UV) using Saremco Print Crowntec hybrid ceramic material. Samples were randomly allocated to two major groups according to post-curing time in a UV polymerization unit (10 min vs. 20 min). Each group was then assigned to two subgroups (*n* = 20 each) for thermocycling treatment (5,000 or 10,000 cycles between 5 °C and 55 °C). All crowns were adhesively cemented on PEEK dies with standardized preparation geometry. Fracture resistance testing was accomplished with a universal testing machine (Instron 5966) using a 5.6 mm steel ball indenter at 0.5 mm/min crosshead speed. Failure modes were examined using scanning electron microscopy SEM. Statistical analysis was conducted using two-way ANOVA (α = 0.05). The 10-minute post-cure group showed slightly greater mean fracture resistance (506.36 ± 58.44 N) than the 20-minute group (492.50 ± 76.06 N), but the difference was not statistically significant (*p* = 0.428). Thermocycling analysis showed that specimens that underwent 5,000 cycles had slightly better fracture resistance (510.00 ± 72.36 N) than those which underwent 10,000 cycles (488.26 ± 62.64 N), but the difference was also not statistically significant (*p* = 0.250). The failure patterns were reported and analyzed with SEM, based on extensions of failures on the axial surface. Although long post-curing times and thermocycling aging exhibited tendencies for decreased fracture resistance, the effects were not statistically significant under the parameters tested. Research in the future should examine the influence of various post-curing intensities and extended aging simulations to further support these results.

## Introduction

The discipline of prosthodontics has seen a paradigm shift with the introduction of digital dentistry^1,2^, and more specifically through the incorporation of additive manufacturing (AM) technologies for the fabrication of definitive dental restorations^3,4^. One of the most notable advances in this area is hybrid ceramic materials - resin-based composites strengthened with high ceramic filler content (40–70% by weight) - which have now been recognized as a new class of dental ceramics under updated ADA guidelines^5,6^. These novel materials retain the mechanical benefits of ceramics while imparting the processing advantages of resins, providing clinicians with restorative options exhibiting enhanced fracture resistance, less abrasive wear against natural teeth, and better marginal adaptation than traditional alternatives^5,7^. The fact that these hybrid ceramics are compatible with newer 3D printing methods like stereolithography (SLA) and digital light processing (DLP) has created new avenues for the streamlined manufacturing of permanent crowns, inlays, and onlays with accuracy and reproducibility^8,9^.

Paramount to the effective translation of these materials to clinical practice lies the optimization of numerous 3D printing parameters that cumulatively dictate the performance of the final restoration^10^. The choice of layer height is a basic consideration, where thinner layers (25–50 μm) provide better surface resolution at the expense of longer printing time and potential error accumulation^11,12^, whereas thicker layers (100 μm) can sacrifice marginal fidelity for increased production efficiency^13^. Of equal significance is build orientation, where angulations from 0° to 45° in relation to the printing platform have been found to maximize mechanical properties by reducing anisotropic behavior and stress concentrations^14,15^. Post-processing procedures, specifically UV curing time (10–30 min) and temperature conditions (40–80 °C), are instrumental in attaining optimal monomer conversion rates, where deviations from ideal may result in either incomplete polymerization or excessive embrittlement^16^.

There is emerging evidence that 3D-printed hybrid ceramic restorations, when processed under optimized conditions, are capable of producing comparable fracture resistance values (450–600 N) to conventionally fabricated lithium disilicate crowns^17^. Long-term durability studies^18–20^ using thermocycling protocols (5,000–10,000 cycles) indicate, however, that the clinical performance of these restorations is strongly reliant on careful parameter calibration since the inherent layer-by-layer construction process can create residual stresses that predispose to microcrack formation under cyclic loading conditions. Notwithstanding these developments, the existing literature exhibits several knowledge gaps, notably with respect to the interaction effects between material composition, printing parameters, and aging resistance, and the absence of standardized post-processing and artificial aging simulation protocols^21,22^.

The introduction of additive manufacturing (AM) in prosthodontics has opened new paradigms in the fabrication of ceramic restorations, but the mechanical reliability of 3D-printed hybrid ceramic crowns (especially their fracture resistance) is still insufficiently studied. Against the backdrop of clinical needs for high-strength, durable restorations, this research systematically addresses three key areas: (1) the influence of crucial AM parameters (e.g., layer thickness, post curing time, and build orientation) on the flexural strength and fracture toughness of hybrid ceramics; (2) their comparative behavior with traditional lithium disilicate and zirconia under simulated mastication (cyclic fatigue) and hydrothermal aging; and (3) the refinement of optimized digital workflows to overcome structural anisotropies intrinsic to the AM processes. By fractographic analysis and Weibull statistics, we measure failure modes and reliability, providing predictive criteria for clinical performance. In conclusion, the null hypothesis posited that no significant differences would be observed between the post-curing times. The evidence-based protocol developed from this study aims to standardize additive manufacturing procedures in prosthodontics, ensuring that 3D-printed crowns meet the biomechanical standards required for definitive restorations while maximizing the advantages of digital dentistry.

## Materials and methods

This in-vitro study used 80 3D-printed Saremco Print CROWNTEC hybrid ceramic (Table 1) crowns designed for a mandibular first premolar. The crowns were cemented onto PEEK dies, which replicated a standard prepared tooth stump. These crowns were randomly allocated to two groups (*N* = 40) according to post-curing time using simple randomization via a random number table. Each group was then divided into two subgroups (*N* = 20) depending on the aging conditions.

**Table 1:** Saremco print CROWNTEC properties.

Density	ca. 1.4–1.5 g/cm3
Flexural strength	≥ 120 MPa Average ≥ 135 MPa
Viscosity	2.500–6.000 mPa
Layer thickness when printing	50 μm
Wavelength 3D printer	385–405 nm
Depth of cure (DIN EN ISO 4049)	≥ 1,5 mm
Flexural strength (DIN EN ISO4049)	> 150 MPa (Mean)
E-Modulus (DIN EN ISO 4049)	> 4000 MPa

All values are as provided by the manufacturer. The density is given as a typical range; values with ‘≥’ represent minimum guaranteed specifications.

A plastic Ivorine mandibular left first premolar was prepared for a full-coverage ceramic crown restoration with a high-speed electric handpiece (200,000 rpm) with air and water cooling and diamond-modified flat-end taper burs (Brassler, Savannah, GA). The preparation consisted of an axial reduction of 1–1.5 mm, a 1 mm rounded shoulder margin, a 1.5 mm occlusal reduction, and a 6-degree taper. The master die was scanned with a Dentsply Sirona inEos X5 scanner. Eighty polyether ether ketone (PEEK) dies were milled to duplicate the prepared master die precisely.

Full-coverage crowns were created with Exocad software, and an automatic margin finder detected the finish line. Standardized occlusal thickness was provided by undercut detection and preparation depth assessment. Cement space and crown thickness were modified accordingly. STL files were processed through nesting software, where the samples were placed at a 45-degree angle with 4 supports per crown. Crowns were 3D printed using hybrid ceramic Saremco Print CROWNTEC material through an Asiga MAX 3D printer that utilized digital light processing (DLP) technology. Printing parameters were 50 μm layer thickness at 35 ± 3 °C (95 ± 3 °F).

To provide homogeneity, the resin was well mixed prior to printing. Polymerization was attained using exposure to UV light, with the platform moving in increments to cure each layer. The printing process created 254 layers per sample, with a thickness of 50 μm each, taking about 30 min. After printing, the samples were retrieved, and dimensions confirmed using a caliper. Post-curing was carried out using a Procure (SprintRay) system, where Group A samples were cured for 10 min, while Group B samples were cured for 20 min.

Samples were embedded in epoxy resin in copper molds, and air bubbles were eliminated with a laboratory vibrator. The resin bases were allowed to set for 24 h for complete polymerization. Supporting structures were gently removed, and finishing of all samples was done with a silicon carbide grinding disc under light contact pressure, following the manufacturer’s instructions. A two-stage polishing procedure was conducted with a pink pre-polishing device at 10,000 rpm and a gray high-gloss polishing device at 8,000 rpm, with light contact pressure for 30 s.

The marginal fit of all samples was evaluated at 3.5× magnification with a sharp No. 5 dental explorer. Adhesive bonding was done according to the APC concept. The intaglio surface was air-particle abraded with 50 μm aluminum oxide at 2 bar pressure for 15 s from a distance of 10 mm. Crowns were bonded using TheraCem^®^, a dual-cure, self-adhesive resin cement with MDP, in accordance with the manufacturer’s instructions. Cement was applied evenly to the internal surface, and crowns were seated under a standardized 2.5 kg load for 5 min with a specially designed cementing device. Samples were light-cured for 2–3 s, excess cement was removed, margins were cleaned, and curing was completed for 40 s. Samples were stored in water at 37 °C for 24 h prior to testing.

Each group was then subdivided into two subgroups (*n* = 20). In subgroups A1 and B1, samples were artificially aged by thermocycling (5,000 cycles, 5–55 °C), in a computer-controlled multifunctional mastication simulator (SD Mechatronik, Feldkirchen-Westerham, Germany). Subgroups A2 and B2 were aged by thermocycling (10,000 cycles, 5–55 °C) in the same simulator.

Fracture resistance was measured with an INSTRON universal testing machine (Instron Industrial Products, Norwood, MA, USA), operated by Bluehill Lite software. A 5 kN load cell captured data^23^. Samples were fastened in the machine’s stationary bottom chamber with tightened screws. Compressive force was applied using a metallic rod with a 5.6 mm spherical tip at a crosshead speed of 1 mm/min. A tin foil sheet was interposed between the rod and the sample to provide evenly distributed stress and to prevent indenter damage. The software captured fracture load, which was detected by an audible crack and an acute decline in the load-deflection graph. Failure load was quantified in Newtons and subjected to statistical analysis.

After the fracture, all the samples were examined for failure extensions under a magnifying lens. Failure patterns were reported and analyzed based on the extensions of failures on the axial surface^24^. Random samples were also analyzed by a scanning electron microscope (SEM) (Quanta 250 FEG, FEI Company, Netherlands) for assessing failure patterns and fractographic analysis. Samples were mounted on an aluminum SEM holder using double-stick carbon tape and coated with a 10 nm gold-palladium layer (EMITECH K550X sputter coater, England).

Numerical data were expressed as means with 95% confidence intervals, standard deviations (SD), and minimum/maximum values. Normality of the data and homogeneity of variance were checked by the Shapiro-Wilk test and the Levene test. Normally distributed data with homogeneous variance were compared by two-way ANOVA, simple effects being compared by using the ANOVA error term. P-values were adjusted by the False Discovery Rate (FDR) procedure, statistical significance being considered at *p* < 0.05. Statistical analysis was conducted by R statistical software version 4.4.2 for Windows.

## Result

Descriptive statistics of fracture resistance (N) values for samples with a 10-minute post-curing time, the mean fracture resistance after 5,000 cycles was 536.00 N (95% CI: 499.60–572.40 N, SD: 58.73 N, range: 450.00–610.00 N), whereas after 10,000 cycles, the mean decreased to 481.67 N (95% CI: 454.93–508.40 N, SD: 47.26 N, range: 400.00–550.00 N). For samples with a 20-minute post-curing time, the mean fracture resistance after 5,000 cycles was 490.00 N (95% CI: 447.83–532.17 N, SD: 77.57 N, range: 400.00–650.00 N), while after 10,000 cycles, the mean was 495.45 N (95% CI: 449.42–541.49 N, SD: 77.89 N, range: 420.00–620.00 N), Table 2.

**Table 2:** Comparisons and summary statistics of fracture resistance (N) with different variables.

Aging cycles	Fracture resistance (*N*) (mean ± SD)		*p*-value
	10 min	20 min	
5,000	536.00 ± 58.73	490.00 ± 77.57	0.110ns
10,000	481.67 ± 47.26	495.45 ± 77.89	0.625ns
p-value	0.065ns	0.843ns	

**Fig. 1:**
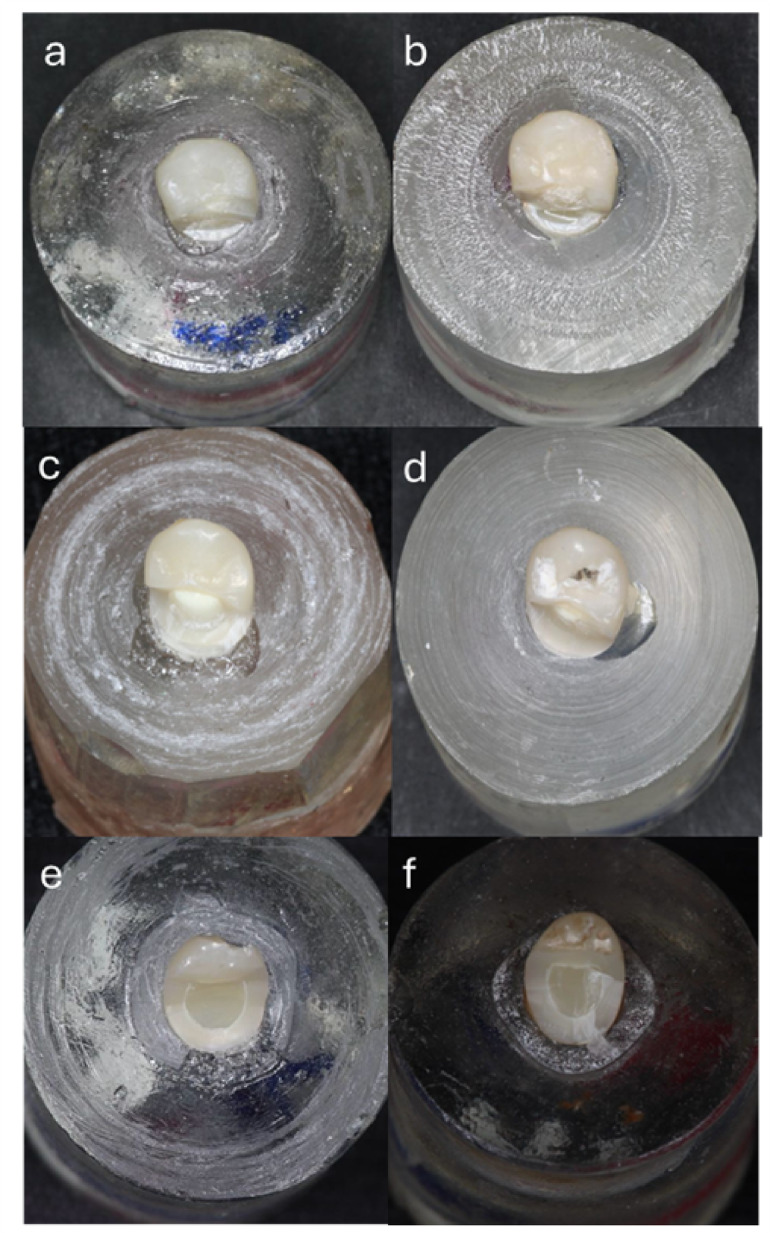
Digital photos representing failure mode analysis of all groups: (**a** & **b**) fracture marginated on the lingual side, (**c** & **d**) fracture extended more proximally, (**e** & **f**) fracture extended till the buccal side.

Regarding failure mode analysis, fracture initiation at the lingual surface was consistent across all groups. While Group A1 fractures were confined to the lingual aspect (Fig. 1a,b), Groups A2 and B2 exhibited lingual-to-proximal propagation (Fig. 1c,d). Group B1 uniquely demonstrated buccal extension in most specimens (Fig. 1e,f), suggesting potential material fatigue or stress concentration differences.

**Fig. 2:**
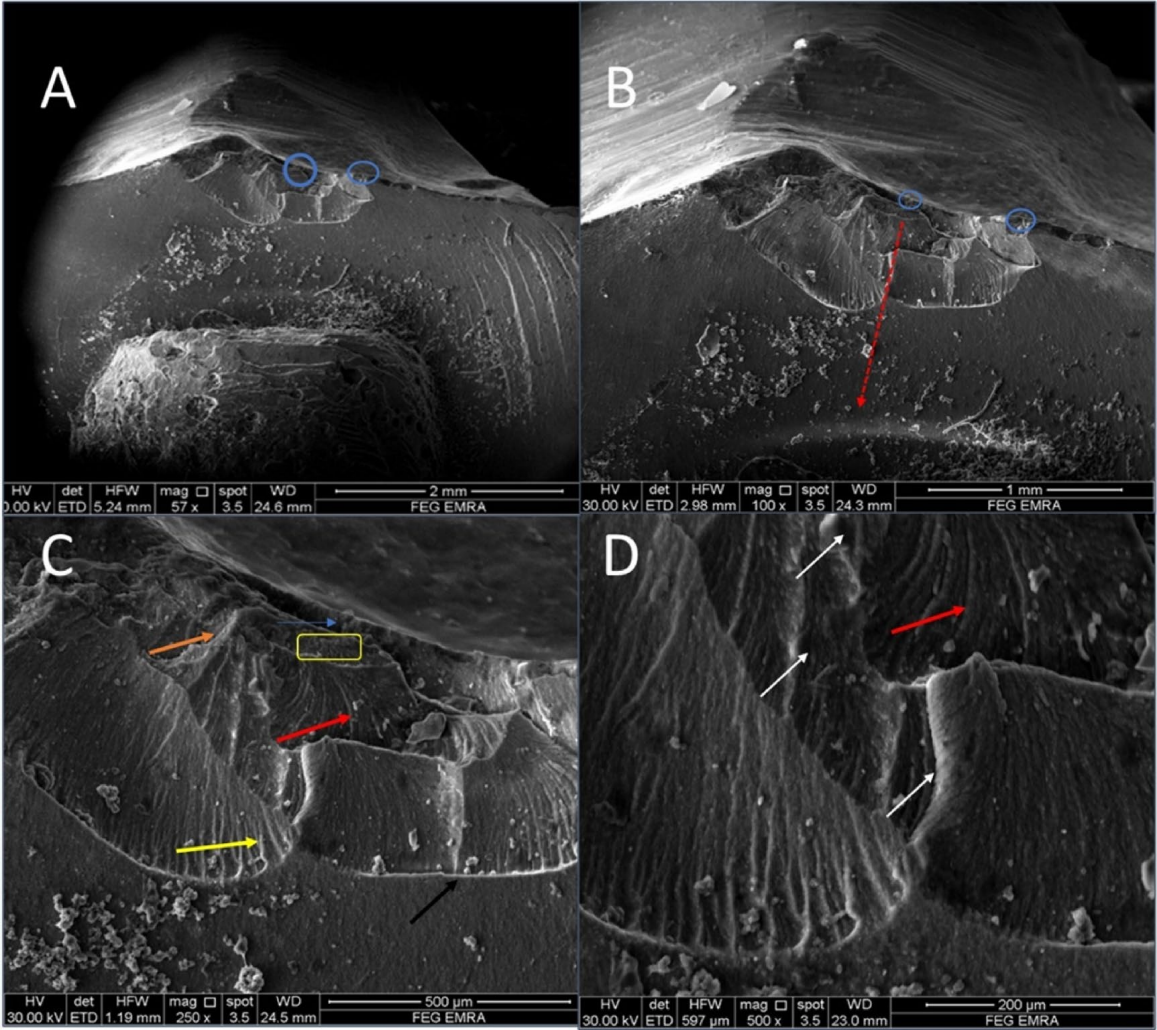
SEM photomicrograph of A1 group showing (57x) multiple crack origins (blue circles, **A**), (100x) crack propagation direction (red dotted line, **B**),(250X) mirror-like zone (blue arrow), mist zone (yellow rectangle), velocity hackles (red arrows), twist hackle (yellow arrow), wake hackle (orange arrow), arrest line (black arrow) and (500x) crack lines (white arrows).

**Fig. 3:**
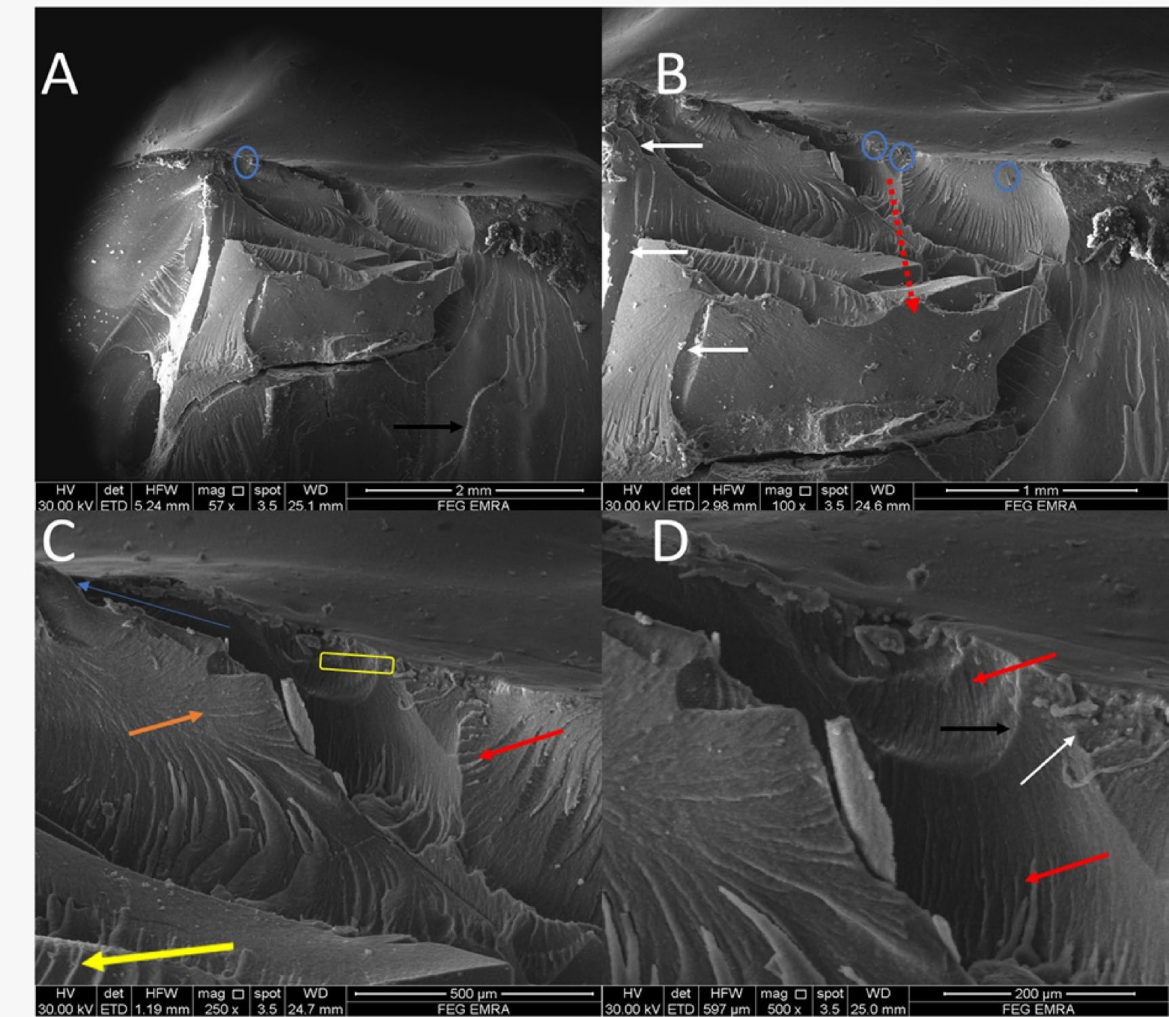
SEM photomicrograph of A2 group showing (57x) multiple crack origins (blue circles, **A**), (100x) radial propagation pattern (red dotted line, **B**),(250X) closer mirror-hackle transition zone (blue arrow), mist zone (yellow rectangle), early formation of velocity hackles (red arrows), twist hackle at small areas (yellow arrow), wake hackle (orange arrow), many arrest line (black arrow) and (500x) multiple secondary intersected crack lines (white arrows).

**Fig. 4:**
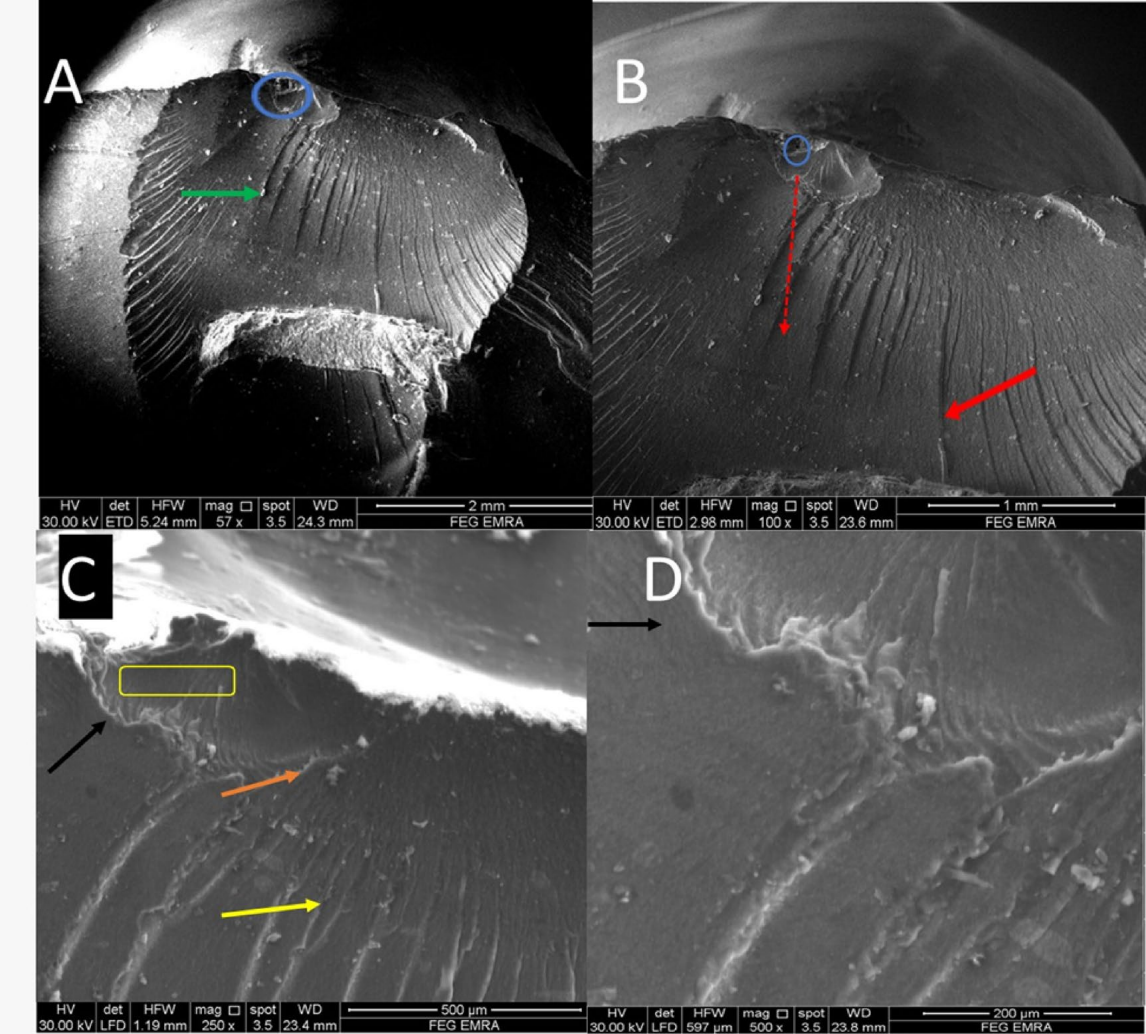
SEM photomicrograph of B1 (57x) showing (**A**) crack origins (blue circle), multiple material artifacts (green arrow), (100x) showed crack propagation direction (red dotted arrow) crack extending in two patterns (**B**), higher magnification (250x) showed mist zone (yellow rectangle), different hackle types (yellow and orange arrow) for twist and wake hackle respectively (**C**), and (black line) representing arrest lines (**D**).

**Fig. 5:**
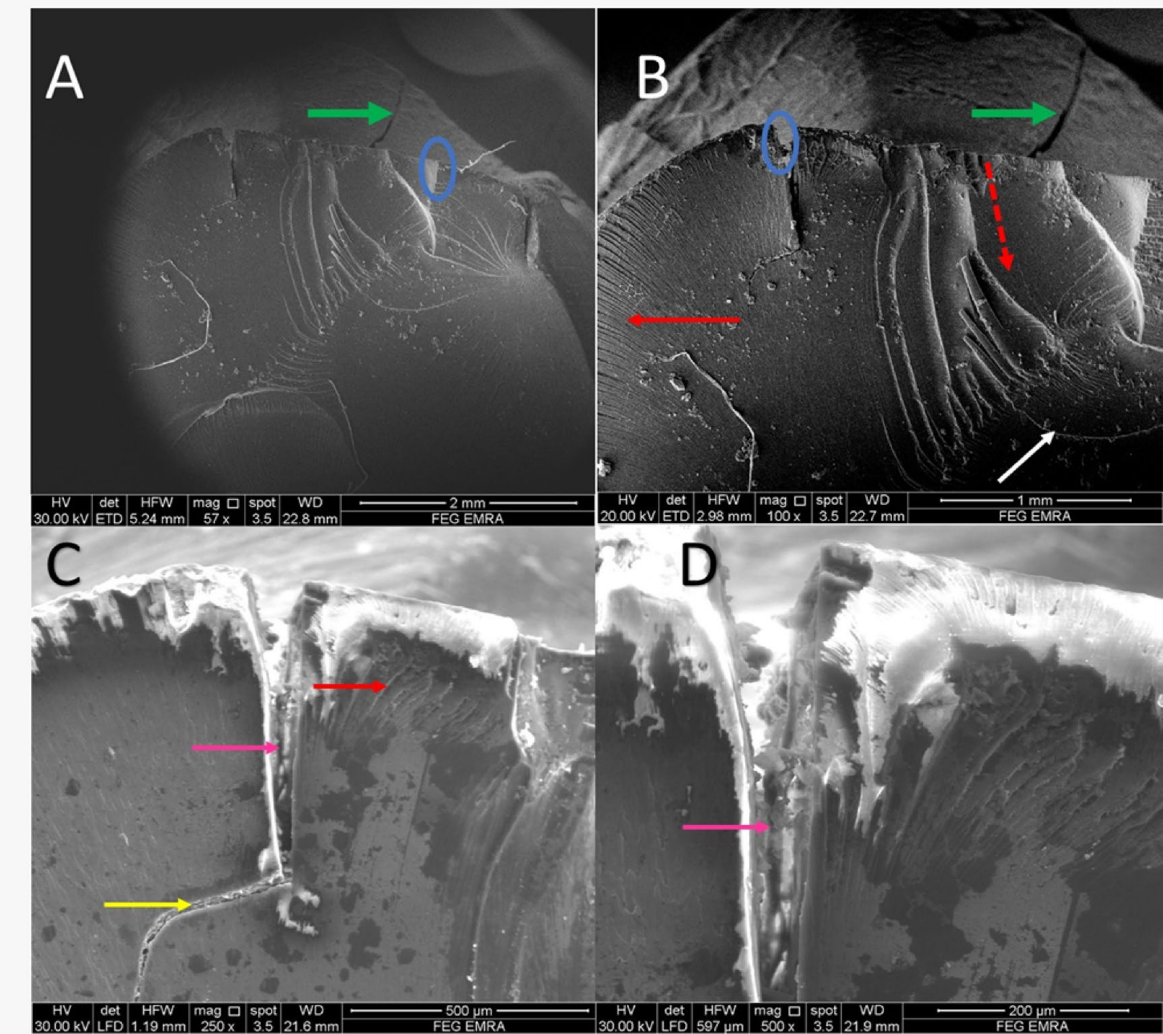
SEM photomicrograph of B2 (57x) showing (**A**) crack origins (blue circle), crescent shape edge fracture at the occlusal surface (green arrow), (100x) showed crack propagation direction (red dotted arrow) and arrest lines (white arrow), higher magnification (250x) showed wake hackle type (yellow arrow) arising from a deep crack (pink arrow) starting occlusal (**C** & **D**).

Fractographic evaluation of the hybrid ceramic crowns revealed consistent fracture initiation patterns across all test groups. At lower magnifications (57–100×), multiple crack origins were consistently identified at the occlusal surfaces, as indicated by blue circles in (Figs. 2, 3, 4 and 5). The specimens exhibited varying degrees of crack propagation, with Group A1 demonstrating minimal radial extension (Fig. 2B), while other groups showed more extensive branching patterns and deeper penetration into the material structure (Figs. 3, 4 and 5). These differences in fracture propagation suggest variations in stress distribution and material response among the experimental groups.

Higher magnification examination (250–500×) provided detailed characterization of fracture surface morphology. All specimens displayed three distinct zones surrounding the crack origins: (1) smooth mirror zones adjacent to the origin (blue arrows), (2) transitional mist areas with increasing surface roughness (yellow rectangles), and (3) well-developed hackle regions. The hackle patterns showed three characteristic morphologies: velocity hackles (red arrows) appearing as deep, divergent grooves; twist hackles (yellow arrows) demonstrating pronounced branching; and wake hackles (orange arrows) exhibiting curved, tail-like striations radiating outward from the origin. Group-specific variations were particularly notable in the hackle patterns, with Group B2 specimens showing more complex, interdigitating fracture lines (Fig. 3) and some groups displaying edge chipping phenomena characterized by crescent-shaped fractures at the crown margins (Fig. 5, green arrows).

The fracture surfaces also contained several features indicative of crack propagation dynamics. Arrest lines (black/white arrows) were observed in multiple specimens, marking points where crack progression was temporarily halted. Material artifacts adjacent to crack origins were noted in some groups (Fig. 4A, green arrow), potentially representing localized defects or inhomogeneities in the material structure. The dimensional characteristics of the mirror zones and the distribution of mist areas varied among groups, suggesting differences in fracture energy and crack propagation rates. These fractographic observations collectively provide insight into the failure mechanisms of the tested hybrid ceramic crowns, with implications for their clinical performance under functional loading conditions.

## Discussion

This study evaluated the influence of post-curing time and thermocycling on the fracture resistance of 3D-printed SAREMCO print CROWNTEC single crowns. The null hypothesis that no significant difference would exist between 10- and 20-minute post-curing times was accepted, as extending the post-curing duration did not significantly enhance fracture resistance. These findings suggest that the mechanical properties of CROWNTEC resin reach optimal polymerization within the initial 10 min of post-curing, with additional time providing no substantial benefit. These could be related to a material with a highly efficient photoinitiator system designed to achieve rapid crosslinking. In such cases, extended curing may become redundant, as the polymer network has already reached a plateau of conversion^8^.

The mean fracture resistance values for the 10-minute (506.36 ± 58.44 N) and 20-minute (492.50 ± 76.06 N) post-cured groups were statistically comparable (*p* > 0.05). The results are consistent with Duarte Jr et al. (2025)^8^, who reported that shorter post-curing durations may be sufficient for achieving adequate polymerization in resins with high photoinitiator efficiency. Similarly, Bonada et al. (2017)^25^ observed no significant differences in flexural strength with varying post-curing times, reinforcing the concept of a polymerization plateau. Additionally, discrepancies with previous literature may be explained by differences in resin formulations, filler content, and light-curing protocols. A post-curing time should be considered material-specific rather than a fixed universal guide^8,25^.

However, conflicting evidence exists: Mahmud et al. (2025)^26^ reported that mechanical properties improved with medium-temperature curing (60–70 °C for 30–60 min), while excessive curing increased brittleness. In fact, a statistically insignificant decrease in mean fracture resistance was observed as more brittle fracture patterns in group 20-min curing under SEM.

Likewise, Kim et al. (2020)^27^ found that flexural strength improved only after 60–90 min of post-curing, depending on resin type. These discrepancies may stem from differences in material formulations, curing protocols, or printer light-source intensity.

Thermocycling (5,000 vs. 10,000 cycles) did not significantly affect fracture resistance (*p* = 0.250). Samples subjected to 5,000 cycles exhibited marginally higher mean fracture resistance (510.00 ± 72.36 N) than those exposed to 10,000 cycles (488.26 ± 62.64 N), but the difference was not statistically significant. This suggests that the material’s mechanical properties stabilize after initial aging, with further thermal stress inducing minimal degradation. These findings align with Gad et al. (2023)^28^ and Korkmaz et al. (2024)^29^, who reported no significant deterioration in 3D-printed resins after extended thermocycling.

The observed stabilization in fracture resistance following thermocycling may be attributed to several underlying mechanisms. Initially, thermocycling induces microcracks and molecular rearrangement within the polymer network^30^, but prolonged exposure leads to structural stabilization as the material reaches a saturated state. Additionally, hygroscopic expansion from water absorption follows a diffusion-limited process, where mechanical properties initially decline but eventually plateau once equilibrium is achieved, minimizing further degradation. The degree of conversion (DC) also plays a critical role^31^, as adequate post-curing promotes sufficient crosslinking and reduces residual monomers, enhancing the material’s resistance to thermal aging^32^.

Fractographic analysis via SEM revealed distinct failure patterns corresponding to different experimental conditions^33^. Specimens post-cured for 10 min exhibited clean fractures with minimal irregularities, suggesting optimal polymer network integrity. In contrast, those post-cured for 20 min demonstrated more brittle fracture patterns, occasionally extending to adjacent surfaces, which may reflect over-curing-induced embrittlement. Furthermore, crowns subjected to 10,000 thermocycles displayed more complex fracture pathways, including mixed lingual and proximal involvement, indicative of progressive fatigue damage from cyclic thermal stress. These observations align with the hypothesis that initial aging induces microstructural changes, while extended exposure leads to crack propagation without further significant loss of mechanical performance.

## Conclusion

The study concludes that post-curing time and aging influence fracture resistance, but the observed differences were not statistically significant under the tested conditions. This research highlights the potential of 3D-printed hybrid ceramics for dental restorations while emphasizing the need for further investigation into optimizing material properties and clinical applications.

- Crowns subjected to a 10-minute post-curing time exhibited slightly higher fracture resistance after 5,000 thermocycling cycles compared to a 20-minute curing time. However, with 10,000 cycles, the fracture resistance of crowns with a 20-minute post-curing time was slightly higher.
- Overall, the differences in fracture resistance due to post-curing time or aging cycles were not statistically significant (*p* > 0.05).
- The results support the viability of 3D-printed hybrid ceramics for permanent restorations, aligning with the physiological demands of the premolar region.

### Clinical significance

The findings suggest that:

- A 10-minute post-cure is sufficient for CROWNTEC crowns, optimizing efficiency without compromising strength.
- Thermocycling within the tested range (5,000–10,000 cycles) does not significantly weaken the material, supporting its clinical durability.

### Limitations

- The experiments were performed under controlled in vitro conditions, which do not perfectly replicate the complex and dynamic intraoral environment.
- Factors such as saliva, bacterial presence, and variable masticatory forces were not accounted for, potentially impacting the clinical relevance of the results.
- The focus on fracture resistance excluded other clinically relevant factors, such as wear resistance, esthetics, or biocompatibility.
- The study examined specific post-curing times and thermocycling protocols, but other variables (e.g., light intensity, wavelength, or alternative aging protocols) were not explored, which could influence outcomes.

## Data Availability

The datasets used and/or analysed during the current study are available from the corresponding author on reasonable request.

## Abbreviations

µ: Mean
SD: Standard deviation
CI: Confidence level
Sig.: Significance level
